# A Micro Rectangular-Shaped Long-Period Fiber Grating Coated With Fe_3_O_4_ Nanoparticle Thin Overlay For Magnetic Sensing

**DOI:** 10.3390/ma8105361

**Published:** 2015-10-19

**Authors:** Sheng-Feng Wang, Chia-Chin Chiang

**Affiliations:** Department of Mechanical Engineering, National Kaohsiung University of Applied Sciences, Kaohsiung 807, Taiwan; 1101403103@gm.kuas.edu.tw

**Keywords:** long-period fiber grating, magnetic sensor, magnetostriction

## Abstract

In this paper, we provide a novel micro rectangular-shaped long-period fiber grating (MRSLPFG) coated with Fe_3_O_4_ nanoparticles as the sensing material and packaged in polydimethylsiloxane (PDMS) for magnetic sensing application. The micro rectangular-shaped grating structures are fully dip coated with the magnetic fluid and heated to form a thin solid film. This thin overlay is used as the sensing media to measure the external magnetic flux density parallel to the optical fiber axis. According to our experimental results, the phenomenon of the transmission loss of the MRSLPFG magnetic sensor was increased monotonically when the external applied magnetic flux density increased. As the external applied magnetic flux density was increased from 0 to 91.10 mT, the resonance attenuation dip of the MRSLPFG increased and the average sensitivity achieved during the experiments was 0.129 dB/mT. We infer that the aforementioned experimental results were due to the magnetostrictive effect exerted on the thin layer of Fe_3_O_4_ nanoparticles, which in turn induced slight longitudinal strains on the micro rectangular-shaped fiber grating structures under different magnetic flux density.

## 1. Introduction

In the last several decades, optical fiber gratings have provoked great interest among scientific researchers working on the development of communication technologies and sensing applications. Optical fiber gratings are devices that are utilized as spectral filters, dispersion compensating components and wavelength division multiplexing systems. Long-period fiber grating (LPFG) consists of periodic refractive index modulations with periods ranging from 100 to 1000 μm [1,2]. This structure promotes light coupling between the core mode and cladding modes to provide an attenuation band in the transmission spectrum [3]. LPFG has many special features: it is light weight, small in size, immune to electromagnetic interference, and highly sensitive. Therefore, it has been commonly applied in the monitoring and measurement of many physical quantities such as magnetic field strength [4,5,6], strain [7], pressure [8], temperature [9], humidity [10], pH [11], and variations in refractive index [12,13].

Among these measurands, the measurement of magnetic field is a most essential aspect of industrial engineering. Magnetic fluid (MF), which is also called ferrofluid, is a promising functional material which possesses versatile magneto-optical properties, including, for example, tunable refractive index, birefringence, dichroism, Faraday effect, thermal-optical effect, magnetochromatics and field dependent transmission. Because MF has diverse magneto-optical effects, it has attracted considerable research interest regarding its use in optical fiber devices such as switches, tunable filters, modulators, magnetic field sensors, and so on [14]. In 2005, Liao *et al.*, presented a tunable optical fiber filter based on a LPFG and the application of a magnetic field to a MF. The motion of the MF jacket, which is controlled by an applied magnetic field, results in a variation of the external index between the MF and air. As a result, the observed center wavelength shift of the attenuation band is 7 nm [15]. Liu *et al.*, proposed a tunable filter based on LPFG coated with MFs as the ambient media. When the MF was subjected to external magnetic fields perpendicularly, the filter’s sensitivity in terms of the center resonant wavelength shift was reported to be 4.45 pm/Oe, and the dip transmission loss was 0.0382 dB/mT [4]. Chieh *et al.*, developed an optical fiber modulator by using MF as a cladding layer. Experimental results showed that the magnetically induced transmission loss of the optical fiber modulator under perpendicular magnetic fields is dominated by the formation of magnetic columns in the cladding MF [16]. In 2009, Tao *et al.* reported on their development of an optical fiber magnetic sensor based on MF, with sensor being composed of a Fabry-Perot interference cavity and a fiber Bragg grating wavelength scanning structure. The sensitivity of that sensor was reported to be approximately 1.5 pm/Oe [5]. Konstantaki *et al.*, reported the magnetic tuning of LPFG through the utilization of water and hydrocarbon-based ferrofluids acting as a cladding layer. When a static magnetic field is perpendicularly applied to this LPFG system, magneto-optical refractive index changes occur in the ferrofluiddic cladding. The sensitivity of the system was shown to be 0.0125 dB/mT [17]. Dai *et al.*, presented a magnetic field sensor that combines an etched fiber Bragg grating (FBG) and MF. The reflected wavelength was changed by altering a magnetic field applied perpendicularly to the axis of the FBG. The wavelength shifts of etched FBG with diameters of 11.3, 10 and 8.5 μm were 25, 46 and 86 pm, respectively. The experimental results showed that the best sensitivity achieved was about 3.44 pm/mT [18]. In a study by Li *et al.*, a magnetic field sensor based on the microfiber knot resonator (MKR) and MF is implemented. The sensitivity of the sensor was reported to be nearly 9.1 pm/mT [19]. Zu *et al.* reported that a magnetic field sensor was configured as a Sagnac interferometer with a magnetic fluid film and a section of polarization maintaining fiber. There were two different case experiments performed in the study. When the magnetic field direction was perpendicular to the light propagation direction, the corresponding sensitivities were 16.7 pm/Oe and 0.3998 dB/Oe. In addition, when the magnetic field was parallel to the light propagation direction, the corresponding sensitivities were −9 × 10^−4^ pm/Oe and 0.0017 dB/Oe [20]. In 2012, Gao *et al.*, designed a magnetic sensor utilizing LPFG written by high frequency CO_2_ laser pulses in a D-shaped fiber and the magneto-optical effects of MF. By immersing the D-shaped LPFG in water-based MF within a capillary, the transmission spectra could be measured while the applied magnetic field intensity was altered. The redshift of the resonant wavelength of the sensor reached as high as 33.46 nm, while the external magnetic field intensity increased to 189.7 mT. Thus, the sensor exhibited a sensitivity of about 176.4 pm/mT [6]. In 2013, Chen *et al.*, fabricated a magnetic field sensor based on the single-mode-multimode-single-mode structure and MF. A demonstration sensor with sensitivities about to 905 pm/mT and 0.748 dB/mT was investigated [21]. In 2015, Zheng *et al.*, demonstrated a magnetic field sensor based on the combination of MF and an optical microfiber mode interferometer. The magnetic field could be measured with a sensitivity of −293 pm/Oe, while the applied magnetic field strength was in the range of 0 to 220 Oe [22]. An optical fiber magnetic field sensor, which was developed by Zhang *et al.*, using a long-period grating coated with MF. They demonstrated that the proposed sensor can maintain a high sensitivity of ~0.154 dB/Gauss at field strength of as low as ~7.4 Gauss. The reported magnetic sensor showed advantage of high sensitivity for low field strength measurement [23].

In this study, we report a new micro rectangular shaped long-period fiber grating (MRSLPFG) and utilize the MF as a dip coated thin overlay to product the magnetic sensor. To begin with, we submerged MRSLPFG into a ferrofluid liquid carried by a PDMS base. Then, we packaged the MRSLPFG in PDMS, which is needed to heat to cure, and cause the MF to develop a thin overlay on the periodic micro rectangular shaped grating structures. The external parallel magnetic field causes the Fe_3_O_4_ thin layer to magnetize, which in turn causes the grating regions to slightly stretch, and this effect could modulate the transmission loss of the MRSLPFG magnetic sensor under distinct magnetic flux density. In 1980, Yariv and Winsor analyzed the possibility of detecting weak magnetic fields by using the magnetostrictive perturbation of optical fibers. A magnetostrictive overlay on optical fiber experienced a longitudinal strain (magnetostriction) when immersed in a magnetic field. Therefore, a magnetostriction induced strain is applied to the optical fibers by the magnetic field. This strain affects the phase delay of a laser light beam propagating in the fiber [24]. The integration of a magnetostrictive material with a LPFG could thus have potential applications in magnetic field sensing. Thomas et al. demonstrated the preparation of metallic glass alloy Metglas 2826 MB based amorphous thin films and examined the magnetostriction on the LPFG based on the Metglas thin film through the attenuation band in transmission spectra [25]. Because of the magnetostriction of a thin metallic overlay, we can obtain the resonant attenuation band spectra by applying various magnetic fields. Hence, the proposed MRSLPFG magnetic sensor can be used as an all-fiber magnetic field sensor or loss-adjustable filter.

## 2. Operating Principle of the Micro Rectangular-Shaped Long-Period Fiber Grating (MRSLPFG) Magnetic Sensor

When the grating period length of a fiber grating ranges from 100 μm to 1 mm, the grating is defined as an LPFG. The LPFG consists of the periodic refractive index change. When light is being propagated in LPFG, the periodic refractive index grating structure will generate the resonant attenuation dip in the spectrum based on the coupled mode theory. In this study, the presented MRSLPFG magnetic field sensor was comprised of an optical fiber sandwiched by a periodic SU-8 photoresist (PR), MF, and PDMS. When an external magnetic field is applied to the MRSLPFG sensor, the periodic grating structures will induce the magnetostrictive effect in the longitudinal direction along the fiber axis and the refractive index variations of the MRSLPFG will be modulated by the magnetostriction behavior of the ferrite material and the strain-optic effect [26]. Thus, the refractive index variations of the MRSLPFG magnetic sensor will be modulated as a periodic square wave distribution along the optical fiber and form the spectrum character of the MRSLPFG. Therefore, the induced strain loading can tune the resonant dip attenuation loss of the MRSLPFG. When this MRSLPFG sensor is subjected to an induced strain, the MRSLPFG reflects the loss variation of the resonant dip. Hence, we can investigate the induced strain variations by monitoring the resonant dip loss. According to the phase matching condition of the coupled mode theory, the wavelength of an LPFG under phase matching conditions can be calculated as follows [26,27]

(1) $$\text{λ}=\text{Λ}(n_{core}^{eff}-n_{clad}^{eff})$$

where Λ is the grating period, $n_{core}^{eff}$ is the effective refractive index of the core mode, and $n_{clad}^{eff}$ is the effective refractive index of the cladding mode. In addition, when light is transmitted in LPFG, the transmission loss can be controlled according to the coupled mode theory for LPFG and the transmission loss has a cosine-squared relationship, which is defined as Equation (2) [26,27]:

(2) $$T=\cos^{2}(\kappa_{co-cl}^{ac}L)$$

where *L* indicates the length of the LPFG, and $\kappa_{co-cl}^{ac}$ is the AC component of the coupling coefficient between the core and the cladding. The transmission loss of the demonstrated MRSLPFG magnetic sensor can be deduced from the AC component of the coupling coefficient between the core and the cladding. By virtue of the periodic variation of the magnetostriction induced strain field, the transmittance will be modulated by the AC coupling coefficient $\kappa_{co-cl}^{ac}$, which is related to the amplitude of changes in the refractive index within the grating structures. When an external magnetic field is applied, the periodic rectangular shaped ferrite grating structures of the MRSLPFG will induce periodic variation in the magnetostriction strain. As a result, the value of $\kappa_{co-cl}^{ac}$ will be altered in accordance with the strain-optics effect. The sensing principle of the MRSLPFG magnetic sensor is based on the monitoring of the transmittance of the MRSLPFG modulated by the external magnetic field. The induced magnetostrictive strain on the MRSLPFG triggers changes to the resonant transmission dip in the MRSLPFG. In this study, the characteristics of the proposed MRSLPFG magnetic sensor are analyzed by exploiting this principle.

## 3. Fabrication Process and Experimental Setup

### 3.1. The Fabrication Process of the Micro Rectangular-Shaped Long-Period Fiber Grating (MRSLPFG) Magnetic Sensor

**Figure 1:**
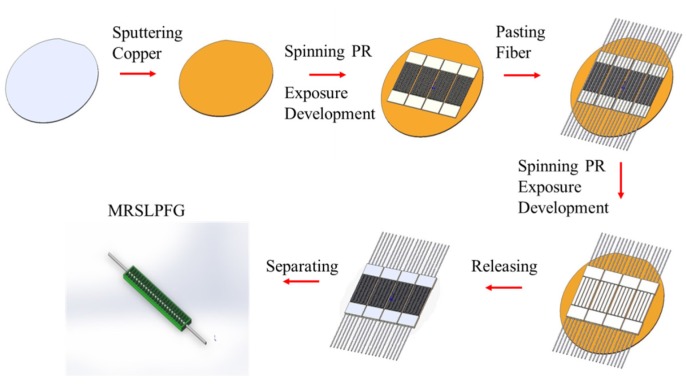
Flowchart of micro rectangular-shaped long-period fiber grating (MRSLPFG) fabrication process.

**Figure 2:**
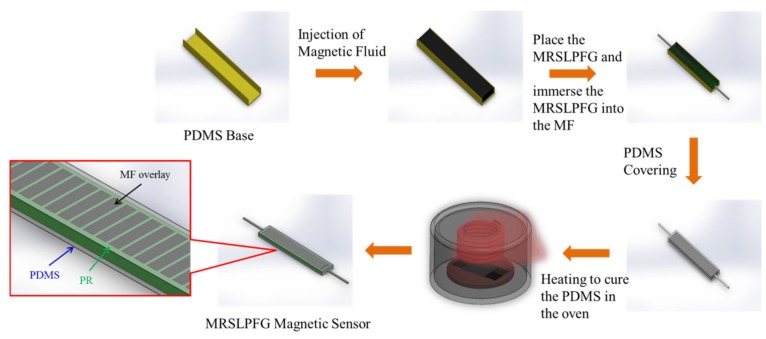
Schematic diagram of the production process of the micro rectangular-shaped long-period fiber grating (MRSLPFG) magnetic sensor.

The photolithography MEMS process was adopted as the procedure for producing the symmetrical sandwiched MRSLPFG magnetic sensor, and the material used in the process included SU-8 3050 negative photoresist (PR), etched single-mode optical fiber, MF and PDMS. Before starting the process, the 4-inch wafer was sputtered with a copper film with a thickness of approximately 200 nm, and the single mode optical fiber was etched in a buffer oxide etching (BOE) solution at 40 °C. The sputtered copper film on the wafer is later used as a released sacrificial layer to allow the sensor to easily separate from the wafer. In the beginning, the production process used a spin coater to evenly coat photoresist onto the surface of the wafer in order to distribute the PR with necessary thickness. Then the coated wafer was placed on a heating plate to carry out the baking operation, the main purpose of which was to allow the vaporization of organic solvents in the PR. After the baking procedure, a single side ultraviolet (UV) light exposing machine was used to carry out the exposure operation. In this process, a 365-nm wavelength UV light was used, and the periodic patterns of optical grating structure were controlled by a plastic mask. After completion of the exposure operation, the wafer was moved onto a hot baking plate to carry out the post exposure bake (PEB) operation in order to eliminate standing waves generated in the exposure operation, and then the hot baking plate was turned off to allow the temperature to slowly cool to room temperature. Finally, after completion of all the aforementioned operations, the wafer was immersed in a solvent developer to remove PR areas that were not exposed to UV light, and the designed bottom periodic structure was obtained. Through the above steps, the first patterned SU-8 3050 PR structure was firmly formed onto the surface of the wafer, and then the single mode optical fiber etched to 35 μm was adhered to the patterned SU-8 structure. The procedures were repeated once again in order to form a periodic structure of sufficient thickness to cover the etched optical fiber. Because the copper sacrificial layer was etched away by the ferric chloride solution, the completed fiber grating on the wafer was then immersed in a ferric chloride solution for the releasing process, whereby the PR layer was separated from the wafer. Figure 1 consists of a diagram of the MRSLPFG fabrication process. Finally, we injected the MF into a PDMS base and located a MRSLPFG in the base to fully dip coat the MRSLPFG. In the meantime, we again used the PDMS to cover the surface of the element, and then the element was placed in an oven to cure at a temperature of 100 °C for 40 min. Upon completion of this curing process, the MF film was turned into a thin solid film coated on the periodic micro rectangular shaped grating structures of the MRSLPFG, and the fabrication of the MRSLPFG magnetic sensor was accomplished. The production process of the MRSLPFG magnetic sensor is illustrated in Figure 2.

### 3.2. Experimental Setup for Magnetic Field Sensing

**Figure 3:**
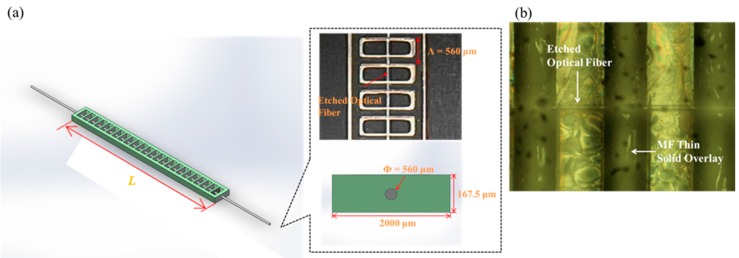
**(a)** Schematic diagram of the MRSLPFG and in-set diagram detailing the dimensions of the grating structures of the MRSLPFG; **(b)** The optic microscope (OM) image of the proposed MRSLPFG magnetic sensor.

**Figure 4:**
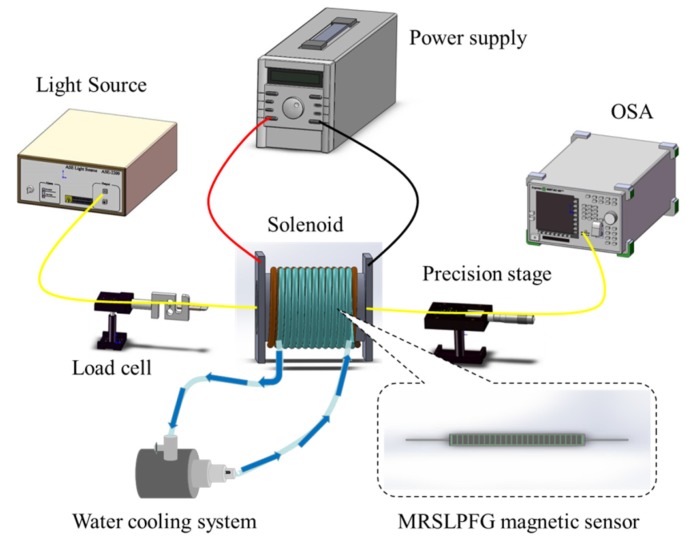
The schematic diagram of experimental setup for the micro rectangular-shaped long-period fiber grating (MRSLPFG) magnetic sensor.

The demonstrated MRSLPFG was fabricated by using photolithography MEMS technique on the single-mode fibers. For this experiment, the grating period Λ of the MRSLPFG was 560 μm, the diameter Φ of the etched fiber was 35 μm, and the length *L* of the grating region was 30 mm. Figure 3a consists of a photograph of the demonstrated MRSLPFG and an in-set diagram detailing its relative dimensions. Figure 3b shows the optic microscope (OM) image of the proposed MRSLPFG magnetic sensor. Fe_3_O_4_ oil-based MF (EMG 901, Ferrotec, Santa Clara, CA, USA) was used in the experiment. This MF is a black-brown opaque liquid which contains 10 nm-diameter ferromagnetic nanoparticles, and the volume concentration is 11.8%. The density of this MF is 1.43 × 10^3^ kg/m^3^. The main purpose of the MRSLPFG magnetic sensor test was to measure the changes of resonant attenuation dip loss of the MRSLPFG when a magnetic field was applied. The experimental setup for magnetic modulation of the MRSLPFG sensor is shown in Figure 4. The experimental equipment included a broadband light source (superluminescent diode, SLD), an optical spectrum analyzer (OSA), a computer, a load cell, a precise translation stage, a power supply, a solenoid and a water cooling circulation system. First, we located the MRSLPFG magnetic sensor into the solenoid and the two ends were fixed on the load cell and precision stage. The MRSLPFG magnetic sensor was placed inside the solenoid and parallel to it. The magnetic field was adjusted by a current generated by the power supply. For every increase of 1 A in the current, the magnetic flux density was increased by 28.47 mT. The current was delivered by the power supply in increments of 0.4 A, increasing from 0 A (0 mT) to 3.2 A (91.1 mT). The solenoid was operated by connecting it to the power supply, and heat was produced when we regulated the magnetic flux density. In order to avoid the thermal effect on the MRSLPFG magnetic sensor, we employed a pump and copper tube to construct a water cooling circulation system that was used to keep the temperature of the electric solenoid steady. During the experimental process, we also monitored the temperature fluctuations by using a thermocouple and maintained the temperature variations within 1.5 °C. Subsequently, the transmission spectra of the MRSLPFG sensor could be monitored and recorded under different magnetic flux densities.

## 4. Results and Discussion

### 4.1. Magneto-Optical Modulation of the Micro Rectangular-Shaped Long-Period Fiber Grating (MRSLPFG) Magnetic Sensor

**Figure 5:**
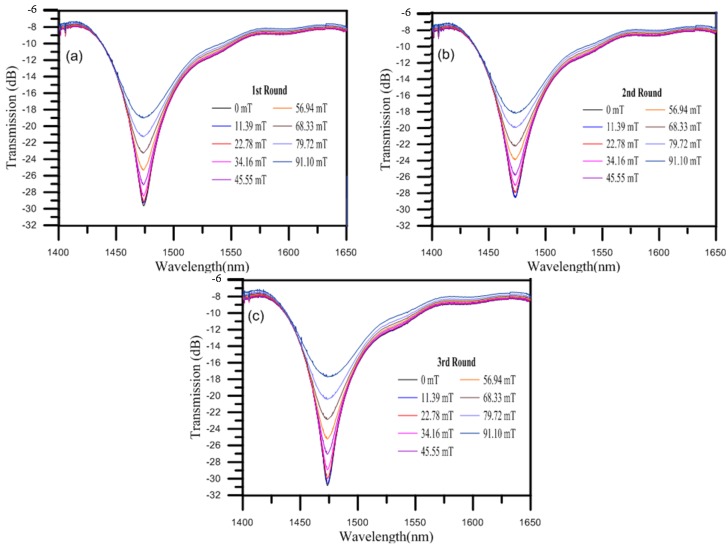
The spectra of the MRSLPFG magnetic sensor under distinct magnetic flux densities. (**a**) Spectra of first round for magnetic field sensing; (**b**) Spectra of second round for magnetic field sensing; (**c**) Spectra of third round for magnetic field sensing.

**Figure 6:**
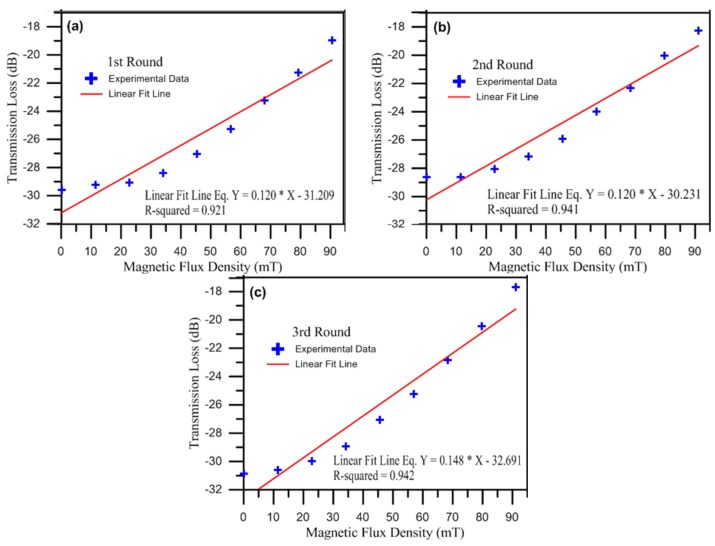
The resonant dips of the transmission loss for the MRSLPFG magnetic sensor under various magnetic flux densities. (**a**) The relationship for the first round of magnetic field sensing; (**b**) The relationship for the second round of magnetic field sensing; (**c**) The relationship for the third round of magnetic field sensing.

The MRSLPFG magnetic sensor is a loss tunable sensor based on periodic refractive index modulation caused by the external axial load. In this study, the periodic refractive index variances of the demonstrated MRSLPFG magnetic sensor are caused by the external loading and the composite soft (PDMS) and hard (SU-8 3050) polymer structures. When a MRSLPFG magnetic sensor is subjected to an axial load, the strain rises and generates a periodic strain field owing to the expanded cross-sectional area of these periodic soft and hard polymer structures. The variations of the periodic refractive index in the fiber form the spectrum characteristic of the MRSLPFG. Hence, the strain can affect the refractive index periodic distribution of the MRSLPFG and generates an attenuation loss dip in the spectrum. Therefore, when a MRSLPFG undergoes external applied strain, the effect of the strain on the MRSLPFG is reflected as loss variation in the resonant dip. Before the magnetic field was applied to the MRSLPFG magnetic sensor, we execute some fixed load on the sensor above the precise translation stage, which was used to firmly hold the optical fiber straight. Additionally, the applied axial load caused the center wavelength of the attenuation band to be 1473.676 nm and caused the dip of the transmission loss to be −29.59 dB. Figure 5 illustrates the relationship between the spectra of the resonant wavelength of the attenuation band loss and the magnetic flux density. As the applied magnetic field was increased, the transmission loss of the resonant dip was gradually increased. This phenomenon is related to the magnetostriction effect and the induced longitudinal slight strain of the periodic ferrite grating structures on the MRSLPFG. The refractive index variation of the entire grating region was affected by the induced magnetostrictive strain on the Fe_3_O_4_ structures of the MRSLPFG. According to Equation (2), we can extrapolate that the induced magnetostriction strain caused by the applied magnetic field of the solenoid resulted in the application of a strain-optic effect on the MRSLPFG. Figure 6 shows the corresponding transmission loss of the MRSLPFG magnetic sensor under magnetic field modulation. When the applied magnetic flux density was altered from 0 to 91.1 mT, the resonant attenuation dips of the MRSLPFG were gradually increased. Figure 6a depicts the relation of the transmission loss under different magnetic flux densities for the first round. As the applied magnetic field increased from 0 to 91.1 mT, the resonant attenuation dip increased monotonically. The values of the transmission loss ranged from −29.59 to −18.971 dB, and the amount of the variance in the transmission loss was 10.619 dB. From these experimental results, we could infer that the Fe_3_O_4_ nanoparticles coated on the micro rectangular-shaped grating structures were magnetized under the external magnetic field and thus caused a magnetostriction effect which, in turn, induced axial strain on the MRSLPFG sensor. Furthermore, the resonant wavelength of the attenuation band rarely shifted during the experiment. We can see that the dip transmission loss was increased monotonically as the magnetic flux density was increased, which can be fitted well by a linear function with a linearity *R*^2^ value of 0.921. The sensitivity was 0.12 dB/mT.

### 4.2. Feasibility of the Proposed Micro Rectangular-Shaped Long-Period Fiber Grating (MRSLPFG) Magnetic Sensor

In order to validate the feasibility of the MRSLPFG magnetic sensor, we performed the experiment three times. After finishing the first round of testing, we turned off the power supply and had to wait about 30 min until the transmission loss of the MRSLPFG magnetic sensor returned close to its original value. The magnetization of the thin overlay coated on the MRSLPFG would gradually be eliminated when the electric solenoid was turned off. Figure 5b shows the second round spectra of the magnetic modulation experiment. The center wavelength of the attenuation band was 1473.177 nm, and the dip of the transmission loss was −28.65 dB. Figure 6b illustrates the relation between the magnetic flux density and the transmission loss for the second round. It shows the same trend that was shown by the first round experiment. The sensitivity was 0.12 dB/mT and linear function with a linearity *R*^2^ value of 0.941. We used the same method to execute the third round experiment. The transmission loss spectra of the magnetic field sensing are shown in Figure 5c. The original value of the transmission loss for the MRSLPFG magnetic sensor, −30.845 dB, was closely restored. According to Figure 6c, the results for the third round of testing resembled those of the first two rounds. The transmission loss increased and the variation in the resonant dip was 13.136 dB as the magnetic flux density was increased from 0 to 91.1 mT. The sensitivity was 0.148 dB/mT and linear function with a linearity *R*^2^ value of 0.942. According to these experimental outcomes, it can be concluded that the proposed MRSLPFG magnetic sensor was successfully modulated by the applied magnetic fields. We also performed the experiment for three rounds in order to validate the characteristics of the MRSLPFG magnetic sensor in terms of feasibility and repeatability. From the results of the experiment, we can calculate the average sensitivity, which is approximately 0.129 dB/mT of the MRSLPFG magnetic sensor for three rounds. That sensitivity was compared, in turn, with the magnetic field sensitivity of the magnetic field sensors developed by Liu *et al.* [4] and Gao *et al.* [6], which were reported to be 0.0382 and −0.1233 dB/mT, respectively. The sensitivity of the MRSLPFG presented in this paper was higher than the sensitivities of those other sensors. It can be concluded that the demonstrated MRSLPFG magnetic sensor has good potential for magnetic sensing applications.

## 5. Conclusions

In this study, we introduce a novel MRSLPFG magnetic sensor for the detection of magnetic field that consists of using Fe_3_O_4_ nanoparticle thin overlay as a sensing material coated on the periodic micro rectangular-shaped grating structures. A MRSLPFG was fabricated by the photolithography MEMS process and combined with a MF and PDMS to create a MRSLPFG magnetic sensor. In addition, experimental results indicated that the MRSLPFG magnetic sensor could successfully detect a magnetic field parallel to the fiber axis that was generated by a solenoid and power supply. According to the results of the experiments, when the MRSLPFG sensor was placed into the coil under magnetic flux density (ranging from 0 to 91.1 mT), the average sensitivity achieved was 0.129 dB/mT. The experimental results also demonstrated that the MRSLPFG magnetic sensor possesses the advantages of feasibility and repeatability. Therefore, the proposed MRSLPFG sensor has the potential to be applied in magnetic field sensing.
